# Functional signature analysis of extreme Prakriti endophenotypes in gut microbiome of western Indian rural population

**DOI:** 10.6026/97320630015490

**Published:** 2019-07-31

**Authors:** Fauzul Mobeen, Vikas Sharma, Tulika Prakash

**Affiliations:** 1 School of Basic Sciences, Indian Institute of Technology Mandi, Kamand 175005, Mandi, Himachal Pradesh, India

**Keywords:** Gut microbiome, Ayurveda, Prakriti, predictive functional profiling, Vata, Pitta, Kapha, imputed metagenomics

## Abstract

Ayurveda is practiced in India from ancient times and stratifies the individuals based on their Prakriti constitution. Advancements in modern science have led to the association of Prakriti with molecular, biochemical, genomic and other entities. We have recently explored the gut microbiome composition and microbial signatures in healthy extreme Prakriti endo-phenotypes. However, their functional potentials are still lacking. The present study includes 63 females (29 Vata, 11 Pitta, and 23 Kapha) and 50 males (13 Vata, 18 Pitta, and 19 Kapha) samples. The predictive functional profiling and organism level functional traits of the human gut microbiome have been carried out in Prakriti groups using imputed metagenomic approach. A higher functional level redundancy is found than the taxonomy across the Prakriti groups, however the dominant taxa contributing to the functional profiles are found to be different. A high number of functional signatures specific to the Prakriti groups were identified in female datasets. Some of the functional signatures were found to be gender specific. For example, a higher abundance of microbes contributing potential pathogenic and stress tolerance related functions was found in Kapha in female and Pitta in male. The functional signatures correlated well with phenotypes and disease predisposition of Prakriti groups.

## Background

The traditional medicinal systems are one of the oldest medicinal systems to diagnose human health and disease conditions and are being practiced worldwide. The Asian region is particularly enriched in these traditional medicinal systems from the ancient times. These systems include Ayurveda (traditional Indian medicinal system), TCM (traditional Chinese medicinal system), SCM (Sasang constitutional medicine), and Kampo medicine (traditional Japanese medicine) [1]. These systems have been proven as extremely useful for the diagnosis, treatment, and preventive measures for disease development. Each traditional medicinal system includes certain underlying fundamental principles in categorizing the constituents which includes doshas, Sasang, Yin-Yang, and Sho in Ayurveda, SCM, TCM, and Kampo medicine, respectively.

Ayurveda is based on the constitution/Prakriti of human individuals which is determined by the prevalent contribution of the doshas viz., Vata (V), Pitta (P), and Kapha (K) in different proportions. The constitution of an individual is known to be decided at birth and is known to be independent of race, ethnicity, and geography. The physiological and psychological characteristics are used to infer the Prakriti type associated with a healthy individual and is carried out by generally experienced Ayurveda doctors. In addition, a few automatic methods including Ayusoft have been developed to classify the Prakriti types of individuals based on their physiological and psychological traits. An imbalance in the proportion of doshas present in healthy individuals may lead to disease conditions [2].

As the Ayurveda discipline is based on ancient practices, various efforts are being made to associate the underlying constitution classification of an individual with the modern molecular mechanisms. There have been several studies to find the Prakriti specific determinants for developing a comprehensive understanding of the constitutions, which includes its association with metabolism, chronic diseases, and genotypes [3]. Further, the immuno-phenotyping based approach has led to the Prakriti specific stratification of samples [4]. Recent advancement in sequencing technologies has led to the establishment of the genetic basis of the Prakriti classification using single nucleotide polymorphism [5]. In addition, the epigenetic analysis of the Prakriti endo-phenotypes has revealed epigenetic signatures [6]. Apart from the genetic level, metabolite level association of the Prakriti groups is also established recently [7]. The metabolites present in the blood plasma are not only contributed by the metabolism occurring in the host but are also contributed and regulated by the gut microbiome [8]. Earlier, we have characterized the taxonomic level gut microbiome differences across the three Prakriti groups viz., Vata, Pitta, and Kapha in male and female datasets [9]. However, the elucidation of their functional potential and the identification of the Prakriti associated functional signatures remains unknown.

An exploration of the functional repertoire of the Prakriti group might contribute in the understanding of the existing differences among the groups with respect to the physiological and psychological traits, host metabolites, immune functions, disease predisposition etc. Towards this, we have carried out a comprehensive predictive functional profiling of the human gut microbiome present across the Prakriti groups in males and females using imputed metagenomics approach. The aim of this study is to predict the potential functional repertoire and to identify the functional signatures and taxa contributions for various important functional traits. The functional signature information thus obtained may broaden our existing knowledge of the contribution of gut microbiome in host phenotype and disease predisposition.

## Methodology

### Organism level functional traits analysis

**Figure 1:**
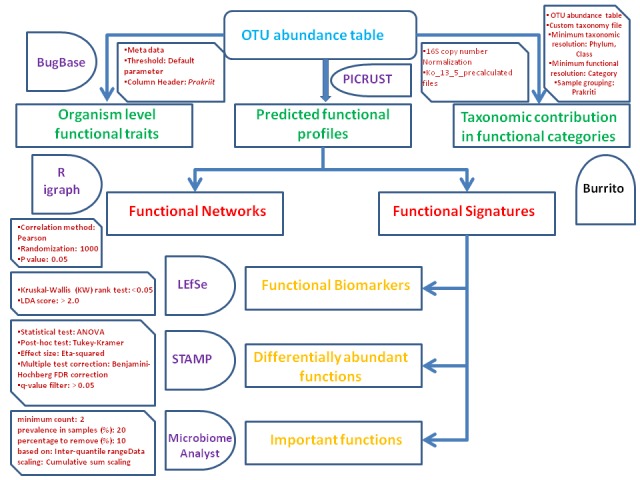
Workflow of the methods used in the imputed metagenomic functional analysis of the Prakriti classified human gut microbiome in male and female datasets.

The present study included 63 females (29 Vata, 11 Pitta, and 23 Kapha) and 50 males (13 Vata, 18 Pitta, and 19 Kapha) samples derived from our previous study [9]. The complete workflow of the methodology is given in Figure 1. The organism level functional traits of the microbiome were calculated using the web based tool "BugBase" (https://bugbase.cs.umn.edu) by employing the OTU abundance table and the associated metadata with the default parameters. The analysis was used to calculate the relative contribution of organism level functional traits including aerobic, anaerobic, facultative anaerobic, gram negative, gram positive, potential pathogens, mobile genetic elements, biofilm formation, and stress tolerance functions across the Prakriti groups in male and female datasets. The relative functional contribution of each taxonomic phylum was also calculated using "BugBase" by employing IMG4, KEGG, and PATRIC databases.

### Imputed metagenomics analysis

Imputed metagenomics of the gut microbiome across the Prakriti groups in male and female datasets were carried out using Phylogenetic Investigation of Communities by Reconstruction of Unobserved States (PICRUST) software [10]. The software performed normalization of the copy number of 16S rRNA based OTUs identified in this analysis against the green genes database (gg_13_5). The prediction of metagenomic functions of the samples were carried out using the KEGG database using ko_13_5_precalculated files. The predicted KEGG functions were collapsed into KEGG level 2 and level 3 pathways. The contribution of the taxa in the functional categories of various Prakriti groups was carried out using the web server "BURRITO" (https://elbospice. gs.washington.edu.shiny/burrito) by providing the OTU abundance table, custom taxonomy file, and metadata. This method normalizes the taxonomic abundance based on the 16S rRNA copy number of the taxa. The gene content corresponding to the taxa were retrieved from PICRUST and the corresponding functions were annotated based on the KEGG database.

### Identification of Functional signatures

The significant functional signature analysis across the Prakriti groups was carried out using the STAMP, LEfSE, and Random Forest [11-13]. The output of PICRUST (.biom file) was converted into STAMP input file (.spf). The identification of significant differentially abundant functions across the Prakriti groups was carried out using STAMP software. The statistical parameters used include Statistical test: ANOVA, Post-hoc test: Tukey-Kramer, Effect size: Eta-squared, Multiple test correction: Benjamini- Hochberg FDR correction and q-value filter > 0.05.

The predicted functional signatures in terms of the KEGG level 2 and level 3 pathways were calculated using the LEfSe software. The differentially existing functions across the Prakriti groups in male and female datasets were identified using a non-parametric Kruskal-Wallis (KW) rank test. The biological consistency was calculated by performing pair-wise tests among the Prakriti groups using the Wilcoxon rank test. Moreover, the effect-size of each differentially expressed function was calculated using the Linear Discriminant Analysis (LDA). We used an alpha value of 0.05 for KW rank sum test and a logarithmic LDA score > 2.0 for effect size estimation. We have performed the LEfSE analysis using strictcriteria on the KEGG level 2 and level 3 pathways. In addition to the strict criteria, due to the high functional redundancy across the Prakriti group microbiomes, we have also used the less-strict criteria on the KEGG level 2 and level 3 pathways for the functional signature analysis.

The important functional attributes of the Prakriti groups were also predicted from the imputed functional profiles using Random Forest module of the Microbiome Analyst with the default parameters. First, this method implemented a low count filter for the KEGG functions including the minimum count and prevalence in the samples as 2 and 20%, respectively. Further, a low variance filter was applied on the KEGG functions, which removed 10% of the less varying KEGG functions based on the inter-quantile range. Finally, data scaling was performed using the cumulative sum scaling (CSS) method. The Random Forest module ranks important functions, which contribute more in the distinction of the Prakriti groups.

### Functional network analysis

The functional networks of the predicted functional profiles across the Prakriti groups were constructed by employing an earlier published method [14]. The method includes identification of the Pearson correlation among the predicted functional profiles and the evaluation of significant correlating functions in the Prakriti groups by randomly shuffling the abundance across all the samples for 10,000 times which gives a p-value. The final network was built by setting a p-value cut off as 0.01. The basic network analysis was performed in the igraph R package [15], which includes the calculation of nodes, edges, density, diameter, centralization, and clustering co-efficient across the Prakriti groups in male and female datasets.

## Results and Discussions

This study presents a functional imputation profiling of the gut microbiomes in 113 healthy individuals' datasets, including 63 females and 50 males. These individuals have been pre-classified with respect to their dominant extreme Prakriti endo-phenotypes, viz. Vata, Pitta, and Kapha [9]. The female dataset includes 29 Vata, 11 Pitta, and 23 Kapha individuals and the male dataset comprises of 13 Vata, 18 Pitta, and 19 Kapha individuals. The taxonomic profiling of these datasets has been carried out previously by our group [9]. In the present study these taxonomic profiles are used to compute the imputed metagenome for all the datasets, which are subsequently used for comprehensive functional analyses. Using these imputed metagenomic profiles; we have explored the multiple ecological and adaptive properties of the healthy human gut microbes with respect to the extreme Prakriti endo-phenotypes.

## Predictive functional profiles of the Prakriti classified human gut microbiome

**Figure 2:**
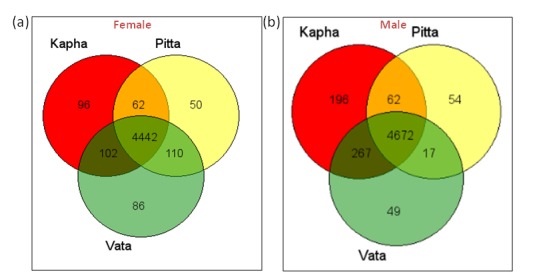
Venn diagrams of the common, shared, and unique KEGG functions across the Prakriti groups viz., Vata, Pitta, and Kapha in (a) female and (b) male datasets.

The predicted functional profiling of the Prakriti classified gut microbiome datasets showed the presence of a total of 4,948 and 5,317 KEGG IDs in the three Prakriti groups in the female and male datasets, respectively. An analysis of the common functions among the Vata, Pitta, and Kapha Prakriti groups revealed a total of 4,442 KEGG IDs in females' (Figure 2a) and 4,672 KEGG IDs in males' (Figure 2b) gut microbiome. Further a comparison of the male and female datasets, irrespective of the Prakriti groups, revealed a total of 4,404 common KEGG functions. At the KEGG functional category level, a highly similar microbiota composition is observed in the Vata, Pitta, and Kapha Prakriti groups in both male and female datasets. These observations indicate that a highly similar functional repertoire of the gut microbiome is present across the Vata, Pitta, and Kapha Prakriti groups and the male and female datasets in terms of the KEGG functions, despite the differences in the overall taxonomic compositions present in these datasets. Functional redundancy is an inherent property of the human gut microbiome, which contributes in its stability and resilience [16]. Besides, the samples included in this analysis were from a similar genetic, dietary, and environmental background.

**Figure 3:**
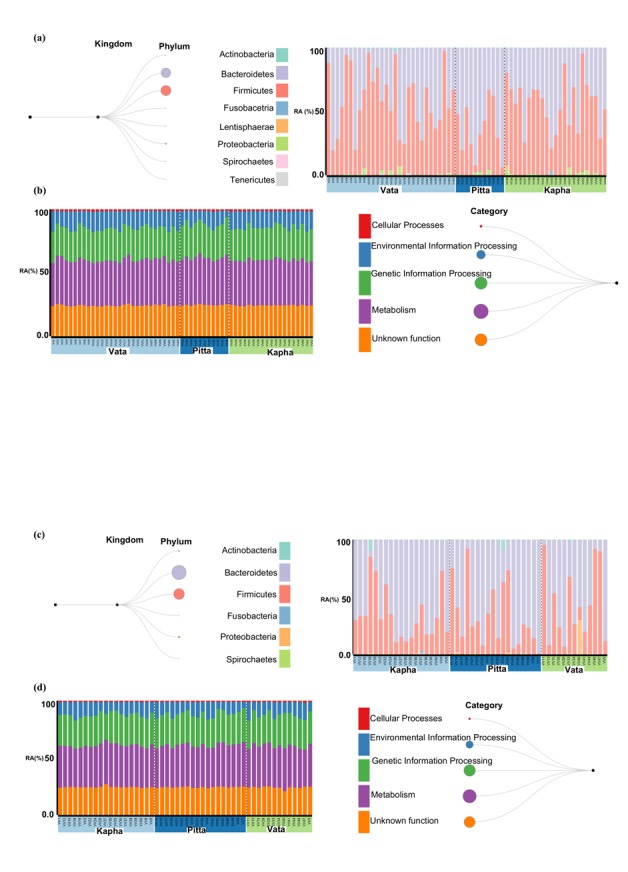
The average taxonomic abundances and their distribution in across Prakriti groups are shown in (a) female and (c) male datasets. The average abundance of functional categories and their distribution across Prakriti group are shown in (b) female and (d) male datasets. RA: Relative Abundance.

These observations indicate that the contributing taxa in the different Prakriti groups and the male and female datasets may be different. Towards this, we have explored the dominant taxa contributing towards a given KEGG functional category in the three Prakriti groups in male and female datasets (Figure 3 and data available with Authors). Our analysis revealed that the phyla Firmicutes and Bacteroidetes were the two most contributing taxa towards the various KEGG functional categories in both males and females datasets. However, a majority of the KEGG functions were contributed by Firmicutes in females and Bacteroidetes in males (Figure 3). In case of females, the ratio in which Firmicutes and Bacteroidetes contribute to the KEGG functions is 2:1 (Table 1), whereas this ratio changes to 1:1 in case of males (Table 1). This implies that as the abundance of Bacteroidetes is increasing in the male samples, their contribution towards various KEGG functional categories is also increasing. Among the KEGG categories, the functions related to "metabolism" were found to be the most abundant followed by "genetic information processing" (Figure 3). The abundances of "cellular process" and "environmental information processing" were found to be lower as compared to the above two functional categories (Available with Authors). In addition, the contribution of the taxa to the various functional categories is proportional to the abundance of the respective KEGG functional category.

Our analysis has revealed a higher contribution of the class Clostridia in all the five KEGG functional categories in Vata and Kapha Prakriti groups in female datasets (Available with Authors). In female Pitta Prakriti group, we observed an increase in the functional contribution of the class Bacteroidia in all the functional categories. Interestingly, in female Pitta Prakriti group the contribution of Bacteroidia was found to be more than Clostridia in "genetic information processing", "metabolism", and "unclassified" functions. In the male datasets, the increase in the functional contribution of the class Bacteroidia over the class Clostridia was found to be more in the Pitta Prakriti group as compared to the Vata and Kapha Prakriti groups. These observations conclude that the contribution of the class Bacteroidia was increasing and that of the class Clostridia was decreasing in the Pitta Prakriti group in both male and female datasets. It is also evident that the contribution of Bacteroidia to the various KEGG functional categories was similar to that of Clostridia in the Pitta Prakriti group (Avaialble with Authors). The reason behind the existence of a lesser difference in the functional contribution of Clostridia and Bacteroidia in the Pitta Prakriti group might be due to the highest microbial evenness (alpha diversity) present within these samples [9].

**Figure 4:**
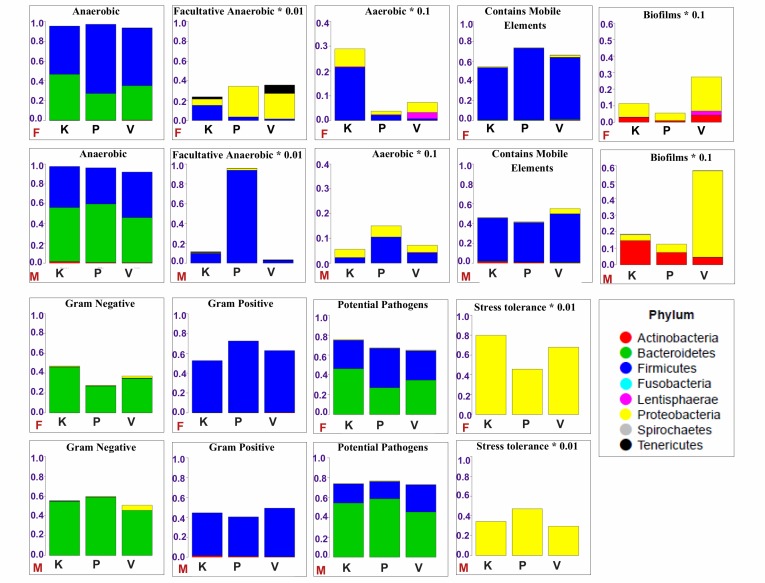
Phylum level contribution in the functional traits in the female and male Prakriti classified human gut microbiome datasets. The functions identified were Anaerobic, Facultative Anaerobic, Anaerobic, Contains Mobile Elements, Biofilms, Gram Negative, Gram Positives, Potential Pathogens, and Stress Tolerance. Vata (V), Pitta (P), Kapha (K), Male (M), Female (F)

It is well known that the Pitta Prakriti group individuals harbour the highest metabolism capacity, which is responsible for the digestion in the gut as well as the cellular and subcellular metabolism [7]. Towards this, we have observed an increased contribution of the class Bacteroidia, which are known to harbour the largest functional repertoire of dietary and host polysaccharide metabolising enzymes [17], in the Pitta Prakriti group irrespective of the gender. The members of the class Clostridia are known to produce SCFAs and have been shown to be beneficial for host due to their function in reduction of intestinal inflammation. A reduction in the abundance of class Clostridia has led to the colonization of potential pathogens in gut and has been implicated in inflammation [18]. In our analysis, a higher abundance of potential pathogenic microbes has been observed in the male Pitta Prakriti group as compared to the other Prakriti groups (Figure 4). An earlier study has already established a higher predisposition potential of the Pitta Prakriti group individuals for inflammation[19].

## Contributing taxa for functional traits

Human gut is well known as an anaerobic environment and the residing microbes are shown to be the major contributors in maintaining this intrinsic property, whereas the role of the oxidative chemistry is also observed in the gut. An overall high abundance of anaerobic microbes is found in healthy human gut and a dysbiosis in the relative abundance of anaerobic and aerobic microbes is observed in various disease conditions [20]. In our analysis, a higher proportion of the anaerobic microbes, as compared to facultative anaerobic and aerobic microbes, is observed in male and female datasets (Figure 4). The anaerobes are found to be primarily affiliated to the phyla Bacteroidetes (B) and Firmicutes (F). However, the abundance patterns of these microbes in the three Prakriti groups are found to be similar in both male and female datasets. A majority of these microbes were taxonomically assigned to Firmicutes in female and Bacteroidetes in male datasets, respectively. The highest relative abundance of the facultative anaerobes was observed in the male Pitta Prakriti group human gut microbiome. A relatively similar abundance of microbes was observed in the female Vata, Pitta, and Kapha Prakriti groups, whereas very low abundance was observed for the facultative anaerobes in the male Kapha and Vata Prakriti groups. A majority of facultative anaerobes in females were taxonomically assigned to the Proteobacteria in the Pitta and Vata Prakriti groups and Firmicutes in Kapha Prakriti group. Further, both male and female Vata and Kapha Prakriti groups showed the presence of phylum Tenericutes in very low abundances, except for female Pitta Prakriti group. In male datasets, the microbes of phylum Firmicutes were the most contributing in the facultative anaerobic traits in all the three Prakriti groups. However, the relative abundance of the Firmicutes in male Pitta Prakriti group was the highest.

A relatively high abundance of aerobic microbes was observed in the Pitta and Kapha Prakriti groups in male and female datasets, respectively (Figure 4). These gender specific differences in the relative abundance of aerobic microbes is not surprising as recent findings have revealed the BMI differences in human gut microbiome in a gender specific manner [21]. Interestingly, a relatively higher abundance of aerobic microbes was observed in the gut microbiome of obese individuals (>27.5kg/m2) as compared to those with normal or lean appearance [21]. In addition, a recent study revealed that most of the Kapha Prakriti group individuals have been found to be associated with a higher BMI (>25 kg/m2) [22]. We also observed Fusobacteria as one of the class contributing various functions only in the Kapha Prakriti group in both male and female datasets. Recently, an enrichment of this class has been observed in the human gut microbiome of obese males [21]. Taken together, these observations indicate towards a possible contribution of aerobic microbes in obesity phenotype. This also indicates that the Kapha Prakriti group individuals may be predisposed to obesity as compared to the Vata and Pitta. A majority of the aerobic microbes in both male and female datasets were taxonomically affiliated to the phylum Firmicutes, except in the female Vata and male Kapha groups, which showed a high contribution of the phylum Proteobacteria. Also, the aerobic microbes affiliated to the phylum Lentisphaerae were only found in the female Vata Prakriti group.

The use of antibiotics has shown to impact the composition of human gut microbiome and usually reduces the microbial diversity. For example, the use of broad-spectrum drugs has shown a preferential expansion of gram negative over gram positive microbes [23]. The gut associated gram positive microbes produce bactericidal compounds, which help host in fighting infections caused by pathogenic bacteria. Previous mice experiments have revealed that antibiotic administration might reduce the commensal gram positive microbes in host gut, thereby, decreasing the bactericidal compounds which leads to a reduced ability of the host to fight pathogenic infections [24]. Thus, the knowledge of the proportion of gram positive and gram negative microbes in the Prakriti human gut microbiome might contribute in the targeted antibiotic treatment regime while reducing its impact on the overall human gut microbiome composition. Interestingly, the Pitta Prakriti group showed the highest abundance of gram negative microbes in the male datasets, whereas, the lowest abundance in the female datasets among the three Prakriti groups (Figure 4). Although both male and females are healthy, however, due to a higher abundance of gram positive microbes in the female individuals with Pitta Prakriti than male, the former may be most affected by application the antibiotic in terms of gut microbiome composition and diversity. A majority of the gram-negative microbes in both male and female datasets were found to be affiliated to Bacteroidetes, whereas, Firmicutes has been found as the most dominant phylum in a large majority of the gram-positive microbes in both male and female datasets.

Human gut harbours not only health-promoting microbes but also those, which are detrimental to health. An analysis of the potential pathogenic microbes' abundance showed relatively smaller differences among the Prakriti groups in male and female datasets (Figure 4).A slightly higher abundance of potential pathogenic microbes is observed in the Kapha and Pitta Prakriti groups in female and male datasets, respectively, as compared to other Prakriti groups. A majority of these microbes were dominantly affiliated to the phyla Bacteroidetes in both male and female Prakriti groups, except in the female Pitta, which showed a high abundance of the phylum Firmicutes. The presence of densely inhabited microbes inside human gut provides a better opportunity for horizontal gene transfer of specific genes through mobile elements for adaptation and survival in the given niche. Our analysis revealed a slightly higher abundance of mobile genetic elements containing microbes in female than the male Prakriti groups (Figure 4). A dominant majority of these microbes were found to be affiliated to the phylum Firmicutes in both male and female Prakriti groups. In addition, Proteobacteria related microbes are found to higher in male and female Vata group as compared to the other Prakriti groups.

The microbes do not occur in isolation, but they co-operate and compete with each other for resource utilization and survival which requires the formation of microbial biofilms. The nutritional and physiochemical environment within human gut also provides a favourable condition for biofilm formation. The biofilms inside the gut have shown several health beneficial advantages which include its role in withstanding the clearance challenges posed by immune effectors and therapeutic antimicrobials [25]. It also provides a platform for bacterial crosstalk to survive the perturbations occurring inside the gut. Further, the microbes present in biofilms are less susceptible to eradication with the antibiotic, environmental factors like pH, and host defence than the other gut bacteria. Thus biofilms provide more robustness in the structure and composition of the microbiome. We observed a comparatively higher abundance of biofilm-forming microbes in the male Prakriti groups than the female Prakriti groups (Figure 4). Among the three Prakriti groups, the Vata group in both male and female datasets is found to have the highest abundance of biofilm forming microbes. A majority of these microbes were taxonomically affiliated with phylum Proteobacteria followed by Actinobacteria. The extracellular polysaccharides of Proteobacteria have been known to play a critical role in biofilm formation [26]. These biofilms finally form an architectural microbial colony through quorum sensing and provide resistance against antibiotics and human immune systems.

The microbes residing in gut experience environmental stress and must overcome it in order to colonize and perform their functions effectively. The operational challenges against gut microbes range from the local environment, including presence of low pH, bile acids, elevated osmolarity, iron limitation, and intermittent nutrient availability, to the host-associated immune factors. To cope up with these conditions, gut microbes harbour several stress tolerance functions. We observed a comparatively higher numbers of stress tolerance functions related microbes in the female than male Prakriti groups (Figure 4). We also detected the highest relative abundances of stress-related functions in Kapha and Pitta Prakriti groups in female and male datasets, respectively. All these microbes were taxonomically affiliated to the phylum Proteobacteria. Stress signalling pathways are found to be more abundant in Proteobacteria than the other phyla. In addition, an increase in the abundance of Proteobacteria has been observed in mouse model of chronic psychosocial stress [27]. We also noticed an opposite abundance pattern of biofilm and stress tolerance functions in the Prakriti groups of male and female, except for female Pitta. This indicates towards the complementing roles of biofilm and stress tolerance related functions in healthy gut microbiome. Survival of pathogenic microbes in human gut must tolerate the environmental factors viz., pH, temperature, and nutrient limitation and induce the stress response against these factors. The Kapha Prakriti group in females harbours the highest stress tolerance functions and may provide the optimum conditions for growth and survival of pathogenic microbes. This is also reflected by the presence of the highest potential pathogens in Kapha Prakriti group in females. We detected a comparatively higher abundance of anaerobic to facultative anaerobic microbes which may lead to lesser oxidative stress tolerance in Vata Prakriti group in males. This corroborates well with the observation of less abundance of oxidative stress related functions in the gut microbiome of underweight males [21].

Although the relative abundances of the phyla Proteobacteria and Actinobacteria are much smaller in healthy human gut, however, the former taxa exhibit important roles in gut-associated functions including biofilm formation and stress tolerance. These functions are crucial for the survival of microbes in harsh gut environment. Despite its low abundance, the phylum Actinobacteria is shown as a keystone taxon in human gut, thus making it important for the existence of a normal healthy microbial assemblage [28]. The keystone taxa consist of those microbes which are involved in the highest number of cross-talk in a given environment. Thus, any perturbations in these microbes are expected to lead to overall dysbiosis in microbial proportions thereby leading to disease conditions.

## Prakriti associated functional signatures

**Figure 5:**
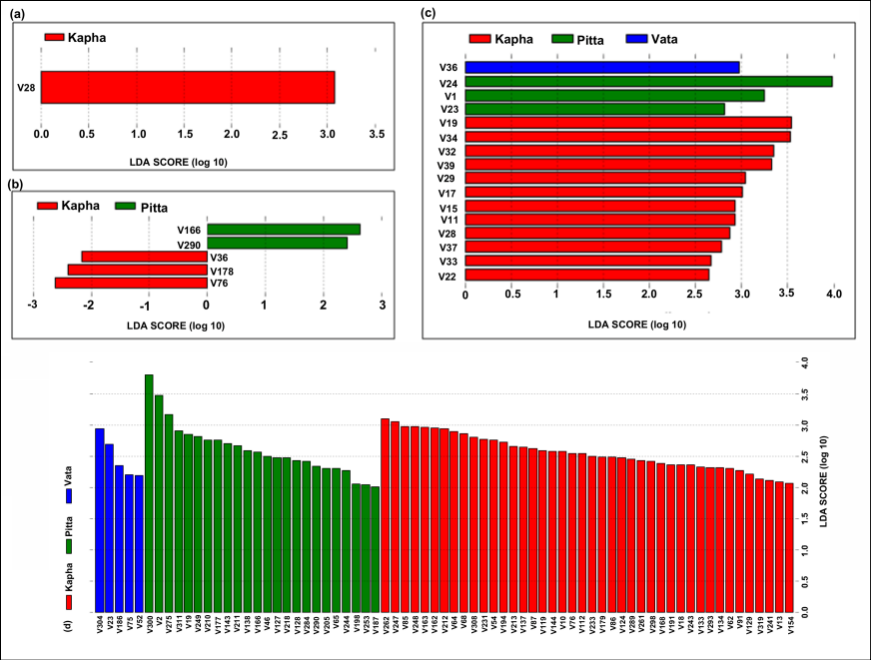
Functional signatures identified in the Prakriti classified female datasets using LEfSe with (a) KEGG level 2 pathways and (b) KEGG level 3 pathways using a more strict-criteria. (c) KEGG level 2 pathways and (d) KEGG level 3 pathways using a less-strict criterion. X axis shows the LDA Scores and Y axis shows the functions. LDA score > 2 is considered as significant. The descriptions of the functions shown on X axis are provided in Table 3. (a) V28: "Metabolism of Other Amino Acids" (b) V166: "Lysine biosynthesis", V290: "Tetracycline biosynthesis", V36: "Biosynthesis of siderophore group nonribosomal peptides", V178: "Mineral absorption", V76: "Cysteine and methionine metabolism" (c) v36: "Signal Transduction", v24: "Membrane Transport", v1: "Amino Acid Metabolism", v23: "Lipid Metabolism", v19: "Glycan Biosynthesis and Metabolism", v34: "Replication and Repair", v32: "Nucleotide Metabolism", v39: "Translation", v29: "Metabolism of Terpenoids and Polyketides", v17: "Folding, Sorting and Degradation", v15: "Enzyme Families", v11: "Digestive System", v28: "Metabolism of Other Amino Acids", v37: "Signaling Molecules and Interaction", v33: "Poorly Characterized", v22: "Infectious Diseases".

**Figure 6:**
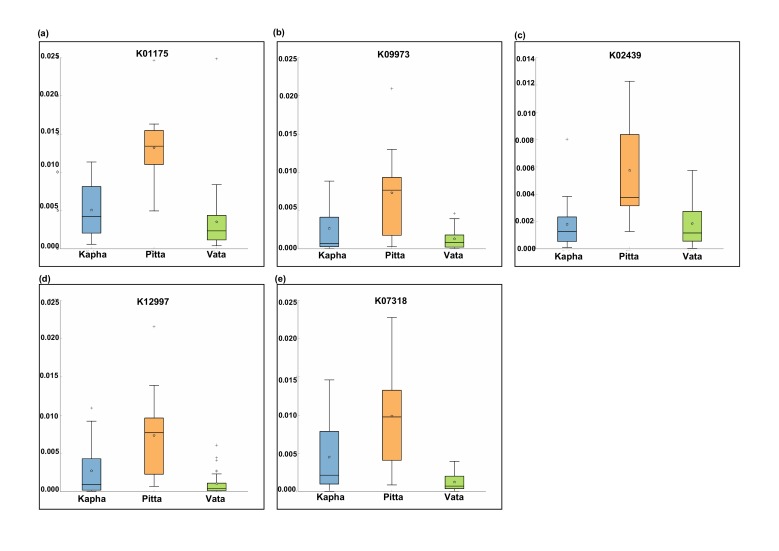
Differentially abundant functions identified using the STAMP (a) unknown function, (b) hypothetical protein, (c) thiosulfate sulfurtransferases, (d) rhamnosyl transferase, and (e) adenine-specific methyl transferase. X axis shows the proportions of the sequences and Y axis shows the female Prakriti groups viz., Vata (V), Pitta (P), and Kapha (K).

**Figure 7:**
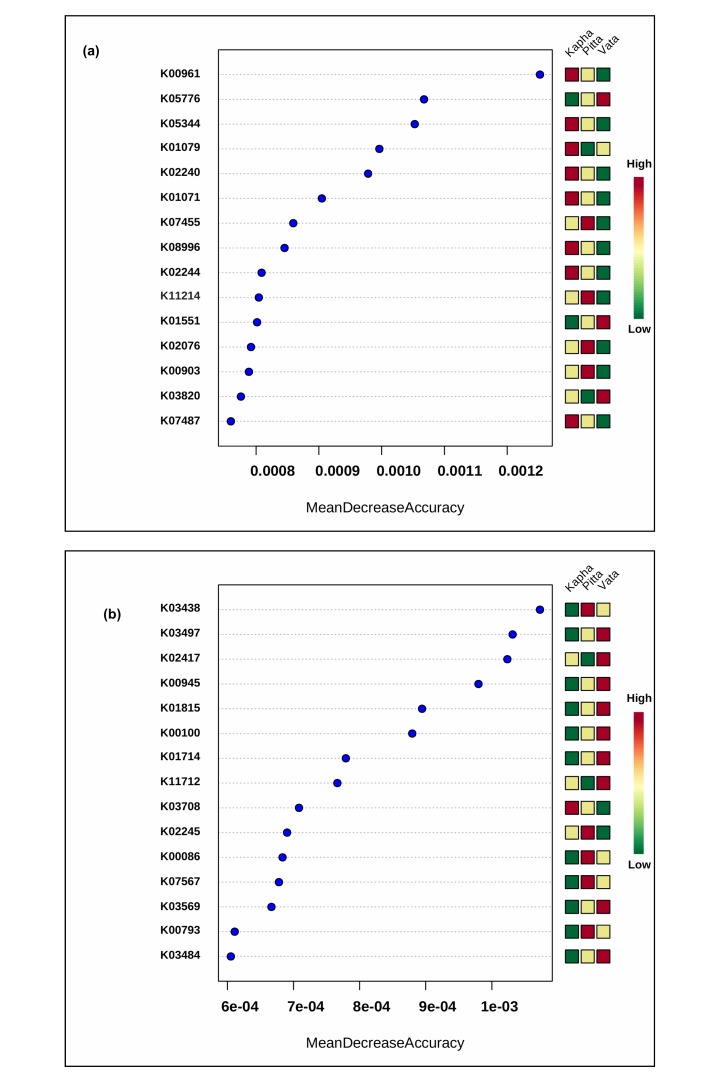
Important functional features identified using the Random Forest analysis in (a) female and (b) male datasets. X axis shows the Mean Decrease Accuracy and Y axis shows the functions. K00961:["DNA polymerase [EC:2.7.7.7]"], K05776:["molybdate transport system ATP-binding protein"], K05344:["glucose-1-phosphate phosphodismutase [EC:2.7.1.41]"], K01079:["phosphoserine phosphatase [EC:3.1.3.3]"], K02240:["competence protein ComFA"], K01071:["oleoyl-[acyl-carrierprotein] hydrolase [EC:3.1.2.14]"], K07455:["recombination protein RecT"], K08996:["putative membrane protein"], K02244:["competence protein ComGB"], K11214:["sedoheptulokinase [EC:2.7.1.14]"], K01551:["arsenite-transporting ATPase [EC:3.6.3.16]"], K02076:["Fur family transcriptional regulator, zinc uptake regulator"], K00903:["protein-tyrosine kinase [EC:2.7.10.-]"], K03820:["apolipoprotein N-acyltransferase [EC:2.3.1.-]"], K07487:["transposase"], K03438:["S-adenosylmethyltransferase [EC:2.1.1.-]", "16S rRNA (cytosine1402-N4)-methyltransferase [EC:2.1.1.199]"], K03497["chromosome partitioning protein, ParB family"], K02417:["flagellar motor switch protein FliN/FliY"], K00945:["cytidylate kinase [EC:2.7.4.14]"], K01815:["4-deoxy-L-threo-5-hexosuloseuronate ketol-isomerase [EC:5.3.1.17]"], K00100:["None"], K01714:["dihydrodipicolinate synthase [EC:4.2.1.52]"], K11712:["two-component system, LuxR family, response regulator DctR"], K03708:["transcriptional regulator CtsR"], K02245:["competence protein ComGC"], K00086:["1,3- propanediol dehydrogenase [EC:1.1.1.202]"], K07567:["TdcF protein"], K03569:["rod shapedetermining protein MreB and related proteins"], K00793:["riboflavin synthase [EC:2.5.1.9]", "riboflavin synthase alpha chain [EC:2.5.1.9]"], K03484:["LacI family transcriptional regulator, sucrose operon repressor"]

**Figure 8:**
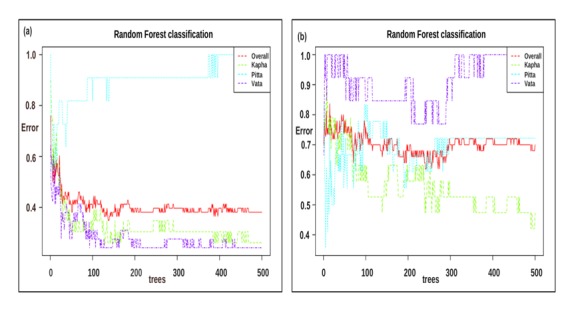
Error plots identified in the Random Forest analysis of human gut microbiome in the Prakriti groups in (a) female and (b) male datasets. X axis shows the number of trees and Y axis shows the corresponding Error.

We have carried out the functional signature identification in predicted functional profiles of gut microbiomes using KEGG functions and KEGG pathways across the Prakriti groups in male and female datasets. The LEfSE analysis using a strict criterion at KEGG level 2 pathways resulted in only one functional signature specific to the female Kapha Prakriti group (Figure 5a). At KEGG level 3 pathways, five functional signatures were identified, including two in Pitta and three in female Kapha Prakriti groups in females (Figure 5b). Upon lowering the criteria for LEfSE analysis to less-strict at KEGG level 2 pathways, sixteen functional signatures were identified in female Prakriti groups including one Vata, three Pitta, and twelve Kapha specific signatures (Figure 5c). At KEGG level 3 pathways, a total of 71 functional signatures were identified which included five Vata, twenty four Pitta, and fourty two Kapha Prakriti group specific functional signatures (Figure 5d and Table 2). Once again no functional signature was identified in the male datasets using the LEfSe analysis. The STAMP analysis resulted in five differentially abundant KEGG functions in the Pitta Prakriti group in females (Figure 6). No differentially abundant functions were identified in the Vata and Kapha Prakriti groups in female datasets and any of the male datasets. The Random Forest analysis was performed to identify the important functions, which can differentiate the Prakriti groups. This analysis resulted into a total of 30 functions in both male and female datasets. We found three, four, and eight functions which were enriched in the Vata, Pitta, and Kapha Prakriti groups, respectively, in female datasets. In the male datasets nine, five, and one enriched functions were identified in the Vata, Pitta, and Kapha Prakriti groups (Figure 7,Figure 8).

## Kapha group specific functional signatures

The KEGG pathway category of "metabolism of other amino acids" belonging to "metabolism" class was identified as the signature for the female Kapha Prakriti group (Figure 5a). In this category, two subcategories, including "glutathione metabolism" and "taurine and hypotaurine metabolism" were related to antioxidant activity. "glutathione metabolism" is a crucial functional subcategory because of the ubiquitous presence of glutathione in every human cell. Besides, it is an important antioxidant for human health which plays a crucial role in homeostasis of cellular oxidative stress. A previous study has demonstrated that the metabolism of this glutathione is governed by gut microbiota in mice model [29]. The KEGG subcategory "taurine and hypotaurine metabolism" is involved in the metabolism of the antioxidant taurine. Tolerance to oxidative stress is an important feature of gut microbes and pathogens in healthy human gut microbiome [30]. Taken together, these functional signatures might be explained by the higher abundance of stress tolerant microbes and potential pathogenic microbes in the Kapha as compared to the other Prakriti groups in females (Figure 4).

The "glycan biosynthesis and metabolism", "enzyme families", "biosynthesis of siderophore group nonribosomal peptides", "cysteine and methionine metabolism", "ubiquinone and other terpenoid-quinone biosynthesis", "glycosphingolipid biosynthesis - ganglio series", "glycosphingolipid biosynthesis - globo series", and "purine metabolism" are the other signature functional categories related to the "metabolism" class in the Kapha Prakriti group in females (Figure 5, and Table 2).Siderophores are lipid compounds which transport iron molecules across cell membrane. Functioning of these compounds is crucial for microbes in ironlimiting conditions as iron is an important component in normal growth and proliferation of microorganisms. Besides, siderophores also act as important virulence factors in pathogenic bacteria [31]. Glycosphingolipids are important membrane constituents which serve signalling roles and have been implicated in host-microbe interactions and microbial pathogenesis [32]. In addition, sphingolipids may be produced by a few opportunistic pathogens. A significant enrichment of "glycosphingolipid biosynthesis - ganglio series" and "glycosphingolipid biosynthesis - globo series" subcategories in the Kapha Prakriti group individuals might result into an increase in the levels of glycosphingolipids in host serum. This is in corroboration with a previous study, which demonstrated an elevated level of serum glycosphingolipid in Kapha Prakriti individuals; however, the study was carried out only on male samples [7].These observations correlate well with the existence of a higher abundance of pathogenic bacteria in the Kapha female group as compared to the other Prakriti groups in females (Figure 4).

Purine metabolism involves the conversion of purine to uric acid by "xanthine dehydrogenase" [33].The abundance of purine metabolism subcategory in the Kapha Prakriti group suggests an increased production of uric acid. This is in corroboration with an earlier study that demonstrated the elevated levels of uric acid in blood serum of the Kapha Prakriti group individuals [34]. The presence of high uric acid in the Kapha Prakriti might predispose these individuals to uric acid mediated diseases. Besides, few KEGG IDs belonging to the "metabolism" class, including glucose- 1-phosphate phosphodismutase and phosphoserine phosphatase, were also found as the functional features of the Kapha Prakriti group of female datasets (Figure 7a). It is known that phosphoserine phosphatase catalyses the production of serine from phospho serine. Serine is also known to act as a precursor for sphingolipid metabolism and contribute in the biosynthesis of purines and pyrimidines [35].The functions related to "ubiquinone and other terpenoid-quinone biosynthesis" have been identified in gut microbiome of patients suffering from a specialized soft tissue cancer viz., colorectal sarcoma [36]. A significantly higher abundance of these functions in the Kapha Prakriti group in females is in corroboration with the previously reported observation that this group might be at a high risk of soft tissue cancer [3].

In the class "genetic information processing" the categories including "folding, sorting and degradation", "replication and repair", and "translation" are identified as the functional signatures of the Kapha Prakriti group (Figure 5d). A higher expression of replication and repair related functions is known in the presence of microbial pathogens in human host [37]. The signature functions viz., "replication and repair" and "translation" are common housekeeping processes and might be more abundant in the Kapha Prakriti group due to a high occurrence of potential pathogens in this group. The competence proteins, viz., ComFA and ComGB of "environmental information processing" are also predicted as the functional features of the Kapha Prakriti group in females (Figure 7a). Microbes have shown to increase the survival during stress conditions by using competence [38]. In addition, these two competence proteins are known to be present in major pathogenic microbes [37]. These observations also corroborate with the presence of high stress-tolerance and potential pathogenic microbes in Kapha Prakriti group (Figure 4).

Only one category of the class "environmental information processing" viz., "signalling molecules and interactions" was predicted as the functional signature of the Kapha Prakriti group in female dataset (Figure 5c). Human gut is known to produce various low molecular weight signalling molecules that drive the functions and interact with the host cellular machinery [39]. These molecules possess the capacity to either turn on or turn off the microbial virulence genes and even host genes. These molecules are also implicated in affecting liver and brain. In addition, these molecules have roles in adiposity and may be implicated in obesity [39]. The "mineral absorption" functions of the "digestive system" is the only predicted functional signature subcategory of the class "organismal systems" in the Kapha Prakriti group of female dataset (Figure 5b). The minerals are supplied from outside to host and are mainly absorbed in gastro-intestinal tract. The gut microbes have been shown to enhance the bio-availability of these minerals in most cases [40]. In addition, a reduction in minerals which are involved in metabolism of glucose and signalling pathway, has been observed in obesity and diabetic conditions. It is known that Kapha Prakriti group individuals are prone to obesity and diabetes [34]. Thus, the identification of significantly high mineral absorption functions in healthy Kapha Prakriti group might provide beneficial effects and prevents this group from obesity and diabetes.

## Pitta group specific functional signatures

Various functions like metabolism, digestion, and energy production are known to be the intrinsic features of the Pitta Prakriti group [3] and all these functions are characterized by gut microbes. Towards this, we have identified "metabolism" related pathways (amino acid and lipid metabolism) along with membrane transporters as the functional signatures of the Pitta Prakriti group females (Figure 5c). The "metabolism" related significant functional subcategories in this group include "lysine biosynthesis", "valine, leucine and isoleucine biosynthesis", "phenylalanine, tyrosine and tryptophan biosynthesis", "arginine and proline metabolism", "histidine metabolism", "tetracycline biosynthesis", "chloroalkane and chloroalkene degradation", and "nitrotoluene degradation" (Figure 5b, Figure 5d and Table 2).

The gut microbiome has been known to produce essential amino acids and interplay roles in amino acid homeostasis. The metabolism of these amino acids might be high in host to maintain homeostasis. Lysine is one of the essential amino acids which is produced in human gut and interplay in host lysine homeostasis [41]. The major source for lysine production in human gut is ammonia derived from the bacterial catabolism of amino acids or urea in intestine [42]. High production of lysine in the Pitta Prakriti group may be due to its intrinsic property of a higher overall metabolism capacity and the presence of significantly higher amino acid metabolism pathways in gut microbiome. A recent study showed that lysine might contribute in the production of butyrate, which serves as an energy source to enterocytes contributing in colonic health [43]. A regulatory effect of lysine has been demonstrated on lipid metabolism [44] and the presence of high lysine biosynthesis might play an important role in the enhanced lipid metabolism capacity in gut microbiome of the Pitta Prakriti group individuals as compared to the other Prakriti groups. To maintain the overall lysine homeostasis a significant enrichment of lysine degrading enzyme "6-amino-2-oxohexanoates" is observed in the blood plasma of the Pitta Prakriti group individuals in males [7]. In addition, we also identified tetracycline biosynthesis as a functional signature of the Pitta Prakriti group which may stimulate lysine production [45]. Apart from lysine, we identified a significant enrichment of biosynthetic pathways of other amino acids including valine, leucine, and isoleucine in Pitta Prakriti group microbiome. Recent studies carried out in blood plasma across Prakriti groups in male also revealed significant presence of degradation pathways of valine, leucine, and isoleucine in the Pitta Prakriti group thereby maintaining the homeostasis of these amino acids [7].

The Pitta Prakriti individuals are known to be more prone to inflammation [9]. The gut bacteria are known to confer inflammatory as well as anti-inflammatory properties. Towards this, we have found some functional signatures for the reduction of inflammation in gut. For example, histidine metabolism, which is known to play an important role in reducing gut inflammation [46] is found as a functional signature of the Pitta Prakriti group individuals in our analysis (Table 2). In addition, we observed a significant presence of "thiosulphate sulphurtransferases" in the Pitta Prakriti group in female (Figure 6c). The thiosulphate sulfurtransferases enzyme is involved in the detoxification of compounds generated through oxidative stress by radiation in liver [47]. It is also known to play a role in the detoxification of H2S in the submucosa and crypts of colon [48]. H2S is known to inhibit butyrate production, which is an important source of energy in the colonic mucosa [49]. H2S produced in gut by sulphate reducing bacteria and host has been shown to induce inflammation. Thus, the presence of a significantly higher abundance of thiosulphate sulfurtransferases might reduce the toxic effects of H2S thus preventing inflammation. Another metabolism related functions viz., Sedoheptulokinase (SHPK), was significantly enriched in the Pitta Prakriti group (Figure 7a) which catalyzes the conversion of sedoheptulose to sedoheptulose-7-phosphate by the expense of ATP. The suppression of pentose phosphate pathway through the activity of sedoheptulokinase has been shown to activate an antiinflammatory M2 macrophage trait [50]. Lipid is one of the most important molecules in the dietary intake which fulfills energy requirements of host. In addition, to providing energy, it provides important bio-molecules and regulates immunity [51]. The higher lipid metabolism pathway identified in this group might contribute in energy, bio molecules, and immunity. The Pitta Prakriti group is known to maintain a high immunity and has been shown to harbour significant enrichment of immune responsive genes [34]. Taken together, our analysis revealed an enrichment of significant functions involved in the anti-inflammatory functions in the Pitta Prakriti group, thus constituting a healthy gut flora.

We identified "signaling and cellular processes" related functional signatures in the Pitta Prakriti group in females which includes "adenine-specific DNA-methyltransferase" (Figure 6e). This enzyme is a member of DNA methyltransferase (DNMTs) family and plays a role in the transfer of methyl groups from S-adenosyl-lmethionine (SAM) to DNA. It is already known that human gut microbiome produces the bioactive compounds which further regulate host epigenome through cross talk [52]. Thus, a significant enrichment of adenine-specific DNA-methyltransferase in gut microbiome may confer more impact on host epigenome. This is in accordance with a recent epigenetic study carried out across the Prakriti groups which revealed the presence of a highest number of gene methylation in blood samples of the Pitta Prakriti group [6]. The ability to remove toxic materials is a characteristic feature of the Pitta Prakriti group [3]. We have identified a significant presence of the functional category related to xenobiotic metabolism in the Pitta Prakriti group individuals. These functional signatures include "chloroalkane and chloroalkene degradation" and "nitrotoluene degradation" (Table 2). This significant occurrence of xenobiotic metabolism may enhance the ability to remove toxic materials in the Pitta Prakriti group individuals. The important functional features identified in the Pitta Prakriti group in male dataset include S-adenosyl-methyltransferase and competence protein ComGC (Figure 7b). The competence protein ComGC represents the virulence properties of microbes [53] and its identification as an important function in the Pitta Prakriti group in male may be due to the presence of the highest potential pathogenic microbes in the Pitta Prakriti group in male.

## Vata group specific functional signatures

We have identified "carbohydrate metabolism" as the signature category belonging to the "metabolism" class as the functional signature of the Vata Prakriti group in female datasets (Figure 6d and Table 2). Carbohydrate is the main constituent of food material consumed in India and is mainly responsible for the production of energy to fulfill host requirements. It is also known to contribute in the majority of short chain fatty acid production in small intestine through fermentation carried out by numerous microbes [54]. The presence of significant enrichment of carbohydrate metabolism in the Vata Prakriti group might lead to the production of more short chain fatty acids and thus contribute in overall wellbeing of host. This is also in accordance with the significant enrichment of butyrate producing microbes in the Vata Prakriti group [9]. These group members are known to be highly active in the processes like movement, waste excretion, and cell division [2]. Thus, the higher energy requirement of the host and the energy consumption through mainly carbohydrate might not allow the accumulation of extra energy and maintain the lean phenotype of this group.

We also found "nitrogen metabolism" from "energy metabolism" and "cyanoamino acid metabolism" from "metabolism of other amino acids" as the signature functional subcategories of the class "metabolism" (Figure 6d and Table 2). The Vata Prakriti group individuals are known to develop neurological disorders and dementia [3]. Recent advancements in human gut microbiome research have suggested a potential role of gut microbes in modulating brain functions through gut-brain axis. The communication between gut and brain are mainly carried out by the modulation of neurotransmitters produced within gut which are mainly nitrogen containing compounds [55]. The changes in concentration of neurotransmitters have been linked to the development of neurological disorder viz., Parkinson's disease. The Vata Prakriti group individuals are known to be more prone to develop neurological disorders. Thus, a high nitrogen metabolism might contribute in the production of these neurotransmitters in Vata gut and help keep a check towards developing neurological disorders.

The "two-component system" of "signal transduction" is the only signature functional subcategory in the class "environmental information processing" (Figure 6d and Table 2). In addition, "signal transduction" is also predicted as a signature category of this functional class (Figure 6c). The identification of significantly higher signal transduction pathway might highlight its role in the cross talk with host [56]. Gut derived molecules have been shown to regulate brain function by building a strong gut-brain axis [55] through a strong signal transduction mechanism. The changes in this mechanism have been linked with the onset of many neurological diseases. Thus, the identification of significant nitrogen metabolism and signal transduction mechanisms in the Vata Prakriti group individuals might highlight their roles in maintaining the overall brain functions and prevent them from neurological disorders.

The "bacterial chemotaxis" belonging to "cell motility" category was the only signature functional subcategory of the class "cellular processes" (Figure 6d and Table 2). Among these signature functions, two-component system is a crucial component of bacteria to sense and respond to the nutrient availability and environmental changes. This system integrates with bacterial chemotaxis which allows bacterial movements in response to the chemical stimulant including nutrients. The process of chemotaxis leads to biofilm formation via auto-aggregation and secretion of autoinducer-2 molecules [57]. To this end, we observed a higher abundance of biofilm forming microbes in the Vata Prakriti group in female dataset which might be attributed to elevated process of bacterial chemotaxis. In the Vata Prakriti group of male dataset 4-deoxy-Lthreo- 5-hexosulose-uronate ketol-isomerase is identified as a functional signature (Figure 7b). Glycosaminoglycans (GAGs) are used by bacteria for their colonization and this property is also observed in probiotic strains found in human gut microbiome [58]. 4-deoxy-L-threo-5-hexosulose-uronate ketol-isomerase performs one of the enzymatic steps of GAG degradation. Thus, the presence of this important colonization function of bacteria in the Vata Prakriti group might be one of the factors for the abundance of biofilm formation related functions in this group in both male and female datasets.

## Basic functional network properties of female and male Prakriti groups

The network analysis in human gut microbiome of female datasets revealed a much higher number of connected nodes in the Kapha followed by the Vata and Pitta Prakriti groups (Table 3). In addition, the Kapha Prakriti group datasets have 1.5 and 2.5 fold higher numbers of nodes as compared to the Vata and Pitta Prakriti group, respectively. In the male datasets, we observed a higher number of nodes in the Pitta Prakriti group than the other two classes (Table 3). The higher numbers of co-occurring functions in the networks might provide robustness to the interactions taking place in the microbiome members during the challenges posed by the external and internal factors like stress and potential pathogens. Consequently, we have identified the Kapha and Pitta Prakriti group datasets in female and male, respectively, to harbour the highest number of stress tolerance functions and potential pathogenic microbes. The network analysis revealed a highly sparse network for the Kapha Prakriti group than the others in females. It also contains more important hubs as shown from the centralization analysis indicating towards its high vulnerability to the external forces of stress and pathogenic microbes. In contrast, the Pitta Prakriti group showed a high density and clustering coefficient which might make it more functionally robust, despite of the presence of low number of nodes and edges. In the male datasets the basic properties of the network were predicted to be high in the Pitta Prakriti group, however, the important hubs and clustering were observed more in the Vata Prakriti group.

In addition to the topological properties, we also identified the top ten interacting nodes in each functional network of male and female Prakriti groups (Table 4). Pyrimidine metabolism is already identified as a functional signature of the Kapha Prakriti group in female. Towards this, thymidylate synthase (TS) is identified as one of the top interacting function in this group in females. This is a critical enzyme for DNA replication and cell growth since it is the only de novo source of thymine nucleotide precursors, including pyrimidine, for DNA synthesis [59]. The other top interacting function in the Kapha Prakriti group is hsp20 family protein, which is a heat shock protein involved in protein folding processes. These proteins are also known to protect the proteins in stress conditions [60]. The "folding, sorting and degradation" pathway harbouring the "hsp20 family protein" is a functional signature identified in the Kapha Prakriti group in females. Another, important function identified in this group is LemA protein, which might be due to the occurrence of maximum potential pathogens in the Kapha Prakriti group in females. This protein has previously been shown to be an important factor in the pathogenicity of certain animal and plant infecting microbes [61]. The ribosomal large subunit pseudouridine synthase D (RluD) has an important functional role in accurate ribosomal assembly and proper functioning [62]. This enzyme is related to ribosome, which is a significant functional signature in the Kapha Prakriti group in females.

In the functional network of the Pitta Prakriti group in females, we identified phosphoserine aminotransferase as one of the most interacting nodes. It is known to play a critical role in the biosynthesis of serine [63]. In addition, we also found peptide methionine sulfoxide reductase as one the most interacting nodes which have revealed its role in the protection of microbes from oxidative damage by reactive nitrogen intermediates [64]. Multidrug resistance protein, viz., multidrug and toxic compound extrusion (MATE) family, is identified as one of the most interacting nodes in the functional network of the Pitta Prakriti group in males. MATE transporters are known to help in extruding drugs thereby contributing in xenobiotic metabolism, which is a characteristic phenotype of the Pitta Prakriti group [65]. In addition, we identified riboflavin kinase as an important interacting node in the functional network of the Pitta Prakriti group in males. This enzyme is known to participate in riboflavin metabolism (Vitamin B2) and production of cofactor flavin mononucleotide (FMN). FMN is an essential cofactor for enzymes involved in the one- and twoelectron oxido-reduction processes, which are important for most of the metabolic energy transformation routes. The Pitta Prakriti group is known to possess the highest metabolism related functions which might involve higher generation and transformation of energy.

## Conclusion

The predictive functional profiles of human gut microbiome across the Prakriti groups in both male and female datasets reveal higher functional redundancy than the taxonomy. The individuals selected for our analysis were from similar genetic, dietary, and environmental background. Thus a higher functional redundancy across the Prakriti groups cannot be ruled out. Interestingly, we observed differences in the dominant contributing taxa in functional profiles across the various Prakriti groups of male and female datasets. The female datasets harboured more number of Prakriti group specific functional signatures as compared to the males. Further, the Prakriti group specific functions also revealed gender specific differences. The predicted functional signatures corresponded well with the phenotypes associated with the Prakriti groups. For example, Pitta individuals are known to be more prone to inflammation. Most of the signature functions identified in this group were belonging to anti-inflammatory functional group in females. Correlating with the highest xenobiotic metabolism potential of the Pitta Prakriti group, functional signatures related to this activity were found in this group. Similarly, the Kapha Prakriti group individuals are found to possess functional signatures associated with obesity and soft tissue cancer, which are known to be high in this group. The Kapha Prakriti group was found to have a higher abundance of functions related to stress tolerance and potential pathogenic activity. The Vata Prakriti group is found to harbour functional signatures related to neurological and stress related disorders. The presented predictive functional profiling of the human gut microbiome will aid in enhancing our understanding of their roles with respect to Prakriti constitutions in healthy males and females. The current study provides first deeper insight into the functional potential of human gut microbiome in the extreme Prakriti and provides its contribution in the phenotypic characteristics and disease predisposition of the host. The current study may also provide baseline study for the assessment of intervention based studies in the Prakriti category to evaluate the effect of the interventions in the maintenance of the better health to reduce the risk factors associated with disease propensity which may also contribute in the development of treatment regime. Thus, the results of this study might also be implicated in the design of early disease preventive regimes of Prakriti groups. In addition, it plays also important role in the understanding of the different responses of the diet and Ayurvedic drugs administered to the Prakriti groups and explain the personalized medicinal systems of Ayurveda.

## Author contributions:

"conceptualization, F.M. and T.P.; methodology, F.M. and V.S.; software, F.M.; validation, F.M.; formal analysis, F.M., V.S. and T.P.; investigation, F.M., V.S., and T.P.; resources, T.P.; data curation, F.M.; writing-original draft preparation, F.M.; writing-review and editing, T.P.; visualization, F.M.; supervision, T.P.; project administration, T.P.; funding acquisition, T.P.".

## Funding:

This research was funded by DBT Ramalingaswamy fellowship of T.P. and The APC was funded by IIT Mandi. F.M. and V.S. acknowledge the Ministry of Human Resource Development (MHRD), India for providing research fellowships.

## Figures and Tables

**Table 1:** Phylum level contribution of the predicted functional categories in female and male datasets

Taxa (female)	Functional Category	Average Attribution of Function	Average Attribution of Total	Total Average Attribution
Actinobacteria	Cellular Processes	0.27	0.01	0.29
	Environmental Information Processing	0.29	0.05	
	Genetic Information Processing	0.28	0.08	
	Metabolism	0.26	0.1	
	Unclassified	0.3	0.05	
Bacteroidetes	Cellular Processes	25.54	1.01	32.25
	Environmental Information Processing	22.3	3.16	
	Genetic Information Processing	34.38	9.93	
	Metabolism	35.26	12.83	
	Unclassified	32.63	5.32	
Firmicutes	Cellular Processes	72.88	3.15	65.94
	Environmental Information Processing	75.58	12.09	
	Genetic Information Processing	63.76	17.87	
	Metabolism	63.05	22.13	
	Unclassified	65.56	10.7	
Fusobacteria	Cellular Processes	0.05	0.01	0.13
	Environmental Information Processing	0.06	0.02	
	Genetic Information Processing	0.03	0.03	
	Metabolism	0.04	0.05	
	Unclassified	0.05	0.02	
Lentisphaerae	Cellular Processes	0.11	0.02	0.59
	Environmental Information Processing	0.1	0.07	
	Genetic Information Processing	0.14	0.17	
	Metabolism	0.15	0.21	
	Unclassified	0.18	0.12	
Proteobacteria	Cellular Processes	1.05	0.04	1.33
	Environmental Information Processing	1.61	0.25	
	Genetic Information Processing	1.34	0.39	
	Metabolism	1.2	0.44	
	Unclassified	1.24	0.21	
Spirochaetes	Cellular Processes	0.06	0.03	0.31
	Environmental Information Processing	0.05	0.07	
	Genetic Information Processing	0.03	0.08	
	Metabolism	0.03	0.09	
	Unclassified	0.03	0.04	
Tenericutes	Cellular Processes	0.02	0	0.04
	Environmental Information Processing	0.01	0.01	
	Genetic Information Processing	0.03	0.02	
	Metabolism	0.01	0.01	
	Unclassified	0.01	0	
Taxa (female)	Functional Category	Average Attribution of Function	Average Attribution of Total	Total Average Attribution
Actinobacteria	Cellular Processes	0.27	0.01	0.29
	Environmental Information Processing	0.29	0.05	
	Genetic Information Processing	0.28	0.08	
	Metabolism	0.26	0.1	
	Unclassified	0.3	0.05	
Bacteroidetes	Cellular Processes	25.54	1.01	32.25
	Environmental Information Processing	22.3	3.16	
	Genetic Information Processing	34.38	9.93	
	Metabolism	35.26	12.83	
	Unclassified	32.63	5.32	
Firmicutes	Cellular Processes	72.88	3.15	65.94
	Environmental Information Processing	75.58	12.09	
	Genetic Information Processing	63.76	17.87	
	Metabolism	63.05	22.13	
	Unclassified	65.56	10.7	
Fusobacteria	Cellular Processes	0.05	0.01	0.13
	Environmental Information Processing	0.06	0.02	
	Genetic Information Processing	0.03	0.03	
	Metabolism	0.04	0.05	
	Unclassified	0.05	0.02	
Lentisphaerae	Cellular Processes	0.11	0.02	0.59
	Environmental Information Processing	0.1	0.07	
	Genetic Information Processing	0.14	0.17	
	Metabolism	0.15	0.21	
	Unclassified	0.18	0.12	
Proteobacteria	Cellular Processes	1.05	0.04	1.33
	Environmental Information Processing	1.61	0.25	
	Genetic Information Processing	1.34	0.39	
	Metabolism	1.2	0.44	
	Unclassified	1.24	0.21	
Spirochaetes	Cellular Processes	0.06	0.03	0.31
	Environmental Information Processing	0.05	0.07	
	Genetic Information Processing	0.03	0.08	
	Metabolism	0.03	0.09	
	Unclassified	0.03	0.04	
Tenericutes	Cellular Processes	0.02	0	0.04
	Environmental Information Processing	0.01	0.01	
	Genetic Information Processing	0.03	0.02	
	Metabolism	0.01	0.01	
	Unclassified	0.01	0	

**Table 2:** Functional signatures identified across the Prakriti groups in female datasets using the LEfSE less-strict criteria at KEGG level 3 pathways.

Kapha	Pitta		Vata	
IDs	Function	IDsFunction	IDsFunction
V262	RibosomeV300	TransportersV304	Two-component system
V247	Purine metabolismV2	ABC transportersV23	Bacterial chemotaxis
V85	DNA repair and recombination proteinsV275	SporulationV186	Nitrogen metabolism
V248	Pyrimidine metabolismV311	Valine,leucine and isoleucine biosynthesisV75	Cyanoamino acid metabolism
V163	Lipopolysaccharide biosynthesis proteinsV19	Arginine and proline metabolismV52	Carbohydrate metabolism
V162	Lipopolysaccharide biosynthesisV249	Pyruvate metabolism
V212	PeptidasesV210	Pentose and glucuronate interconversions
V64	Chaperones and folding catalystsV177	Methane metabolism
V68	ChromosomeV143	Histidine metabolism
V308	Ubiquinone and other terpenoid-quinone biosynthesisV211	Pentose phosphate pathway
V231	Pores ion channelsV138	Glyoxylate and dicarboxylate metabolism
V54	Carbon fixation pathways in prokaryotesV166	Lysine biosynthesis
V194	One carbon pool by folateV46	C5-Branched dibasic acid metabolism
V213	Peptidoglycan biosynthesisV127	Glycerolipid metabolism
V137	GlycosyltransferasesV218	Phenylalanine, tyrosine and tryptophan biosynthesis
V87	DNA replication proteinsV128	Glycerophospholipid metabolism
V119	General function prediction onlyV284	Sulfur relay system
V144	Homologous recombinationV290	Tetracycline biosynthesis
V10	Amino acid related enzymesV205	Pantothenate and CoA biosynthesis
V76	Cysteine and methionine metabolismV65	Chloroalkane and chloroalkene degradation
V112	Folate biosynthesisV244	Protein kinases
V233	PrenyltransferasesV198	Other transporters
V179	Mismatch repairV253	RNA transport
V86	DNA replicationV187	Nitrotoluene degradation
V124	Glutathione metabolism
V289	Terpenoid backbone biosynthesis
V261	Riboflavin metabolism
V298	Translation factors
V168	Lysosome
V191	Nucleotide excision repair
V18	Arachidonic acid metabolism
V243	Protein folding and associated processing
V133	Glycosphingolipid biosynthesis - ganglio series
V293	Toluene degradation
V134	Glycosphingolipid biosynthesis - globo series
V62	Cellular antigens
V91	Drug metabolism - other enzymes
V129	Glycine, serine and threonine metabolism
V319	Vitamin B6 metabolism
V241	Protein digestion and absorption
V13	Aminobenzoate degradation
V154	Isoquinoline alkaloid biosynthesis

**Table 3:** The basic network properties in the Vata, Pitta, and Kapha groups in female and male functional gut microbiome

Network Property (female)	Vata	Pitta	Kapha
Nodes	2030	1338	3285
Edges	25506	18496	291052
Density	0.006	0.103	0.026
Diameter	16	9	32
Centralization	0.041	0.07	0.09
Clustering coefficient	0.262	0.372	0.355
Network Property (Male)	Vata	Pitta	Kapha
Nodes	2050	2117	973
Edges	35112	43704	9220
Density	0.008359104	0.009756289	0.009748815
Diameter	17	22	8
Centralization	0.05071883	0.07250859	0.08086928
Clustering coefficient	0.3448578	0.2802401	0.29

**Table 4:** The top ten interacting nodes and the corresponding functions in functional networks of the Prakriti groups in females and males

Vata	Number of egdes	Function	Pitta	Number of egdes	Function	Kapha	Number of egdes	Function
K01491	192	["methylenetetrahydrofolate dehydrogenase (NADP+) / methenyltetrahydrofolate cyclohydrolase [EC:1.5.1.5 3.5.4.9]"]	K01938	216	["formate--tetrahydrofolate ligase [EC:6.3.4.3]"]	K00560	770	["thymidylate synthase [EC:2.1.1.45]"]
K02914	176	["large subunit ribosomal protein L34"]	K00831	204	["phosphoserine aminotransferase [EC:2.6.1.52]"]	K13993	740	["HSP20 family protein"]
K00820	176	["glucosamine--fructose-6-phosphate aminotransferase (isomerizing) [EC:2.6.1.16]"]	K02168	204	["high-affinity choline transport protein"]	K03744	738	["LemA protein"]
K01952	176	["phosphoribosylformylglycinamidine synthase [EC:6.3.5.3]"]	K07447	200	["putative holliday junction resolvase [EC:3.1.-.-]"]	K06180	730	["ribosomal large subunit pseudouridine synthase D [EC:5.4.99.12]", "23S rRNA pseudouridine1911/1915/1917 synthase [EC:5.4.99.23]"]
K01874	176	["methionyl-tRNA synthetase [EC:6.1.1.10]"]	K07456	200	["DNA mismatch repair protein MutS2"]	K01897	726	["long-chain acyl-CoA synthetase [EC:6.2.1.3]"]
K00057	170	["glycerol-3-phosphate dehydrogenase (NAD(P)+) [EC:1.1.1.94]"]	K02992	200	["small subunit ribosomal protein S7"]	K01155	726	["type II restriction enzyme [EC:3.1.21.4]"]
K00134	170	["glyceraldehyde 3-phosphate dehydrogenase [EC:1.2.1.12]"]	K03550	200	["holliday junction DNA helicase RuvA [EC:3.6.4.12]", "holliday junction DNA helicase RuvA"]	K05592	720	["ATP-dependent RNA helicase DeaD [EC:3.6.4.13]", "ATP-dependent RNA helicase DeaD"]
K02871	166	["large subunit ribosomal protein L13"]	K02871	198	["large subunit ribosomal protein L13"]	K07098	720	["None"]
K00791	152	["tRNA dimethylallyltransferase [EC:2.5.1.75]"]	K07082	196	["UPF0755 protein"]	K00789	720	["S-adenosylmethionine synthetase [EC:2.5.1.6]"]
K03770	150	["peptidyl-prolyl cis-trans isomerase D [EC:5.2.1.8]"]	K09812	194	["cell division transport system ATP-binding protein"]	K06966	718	["None"]
Vata	Number of egdes	Function	Pitta	Number of egdes	Function	Kapha	Number of egdes	Function
K03119	242	["taurine dioxygenase [EC:1.14.11.17]"]	K03327	378	["multidrug resistance protein, MATE family"]	K11749	308	["regulator of sigma E protease [EC:3.4.24.-]"]
K01782	238	["3-hydroxyacyl-CoA dehydrogenase / enoyl-CoA hydratase / 3-hydroxybutyryl-CoA epimerase [EC:1.1.1.35 4.2.1.17 5.1.2.3]"]	K01993	278	["HlyD family secretion protein"]	K12267	304	["peptide methionine sulfoxide reductase msrA/msrB [EC:1.8.4.11 1.8.4.12]"]
K00249	234	["acyl-CoA dehydrogenase [EC:1.3.99.3]"]	K07101	272	["None"]	K09155	302	["hypothetical protein"]
K07302	228	["isoquinoline 1-oxidoreductase, alpha subunit [EC:1.3.99.16]"]	K06950	266	["uncharacterized protein"]	K12410	292	["NAD-dependent deacetylase [EC:3.5.1.-]"]
K03862	228	["vanillate monooxygenase [EC:1.14.13.82]"]	K02065	260	["putative ABC transport system ATP-binding protein"]	K02343	292	["DNA polymerase III subunit gamma/tau [EC:2.7.7.7]"]
K01061	228	["carboxymethylenebutenolidase [EC:3.1.1.45]"]	K02536	256	["UDP-3-O-[3-hydroxymyristoyl] glucosamine N-acyltransferase [EC:2.3.1.-]"]	K00831	290	["phosphoserine aminotransferase [EC:2.6.1.52]"]
K05566	226	["multicomponent Na+:H+ antiporter subunit B"]	K02314	254	["replicative DNA helicase [EC:3.6.4.12]", "replicative DNA helicase [EC:3.6.1.-]"]	K02968	288	["small subunit ribosomal protein S20"]
K05567	218	["multicomponent Na+:H+ antiporter subunit C"]	K11753	252	["riboflavin kinase / FMN adenylyltransferase [EC:2.7.1.26 2.7.7.2]"]	K03086	288	["RNA polymerase primary sigma factor"]
K05569	218	["multicomponent Na+:H+ antiporter subunit E"]	K00912	252	["tetraacyldisaccharide 4'-kinase [EC:2.7.1.130]"]	K07005	288	["None"]
K03638	218	["molybdenum cofactor biosynthesis protein B"]	K12507	250	["acyl-CoA synthetase [EC:6.2.1.-]"]	K02954	286	["small subunit ribosomal protein S14"]
